# A case of breast cancer in the axillary tail of Spence – enhanced magnetic resonance imaging and positron emission tomography for diagnostic differentiation and preoperative treatment decision

**DOI:** 10.1186/1477-7819-11-217

**Published:** 2013-09-03

**Authors:** Mai Okubo, Keiichiro Tada, Takayoshi Niwa, Kotoe Nishioka, Eiichi Tsuji, Toshihisa Ogawa, Yasuyuki Seto

**Affiliations:** 1 Department of Breast and Endocrine Surgery, The University of Tokyo Hospital, Japan 7-3-1 Hongo, Bunkyo-ku, Tokyo, 113-8655, Japan

**Keywords:** Breast neoplasms, Axillary tail of spence, MRI, PET

## Abstract

**Background:**

The management of cancer in the axillary area depends on the etiology of the tumor.

**Case Report:**

A 37-year-old woman presented with a 2 cm mass in the axillary fossa. Core needle biopsy revealed adenocarcinoma. There were no abnormal breast findings on physical examination, mammography, or ultrasonography. However, enhanced magnetic resonance imaging (MRI) and positron emission tomography (PET) showed a segmentally-distributed, abnormal area in the upper-outer quadrant, continuous with the axillary mass. Samples of this area obtained by vacuum-assisted biopsy showed intraductal carcinoma. These findings indicated that the axillary lesion was a part of primary breast cancer originating from the axillary tail. Based on these results, the patient underwent total mastectomy with sentinel lymph node biopsy. Pathological examination of the specimen showed invasive ductal carcinoma accompanied by intraductal carcinoma extending up to 8.5 cm. Our case suggests that enhanced MRI and PET can provide useful preoperative information for the management of axillary breast lesions.

## Background

Cancerous tumors located in the axillary fossa can be the result of several processes, including occult breast cancer with axillary lymph node metastasis, ectopic breast cancer, axillary tail breast cancer, or metastatic lymphadenopathy originating from non-breast tissue. Management of this lesion is dependent on the etiology. Preoperative evaluation, therefore, is important. In this article, we report a case of a cancerous axillary lesion originating from the axillary tail. Positron emission tomography (PET) and enhanced magnetic resonance imaging (MRI) provided useful information in relation to management.

## Case presentation

### Case

A 37-year-old woman presented to our hospital with a right axillary mass. Fourteen months prior to her presentation at our institution, she presented to a different breast clinic with the same complaint. However, she was told that there were no abnormal findings based on normal screening mammography and ultrasonography of the breast.

**Figure 1:**
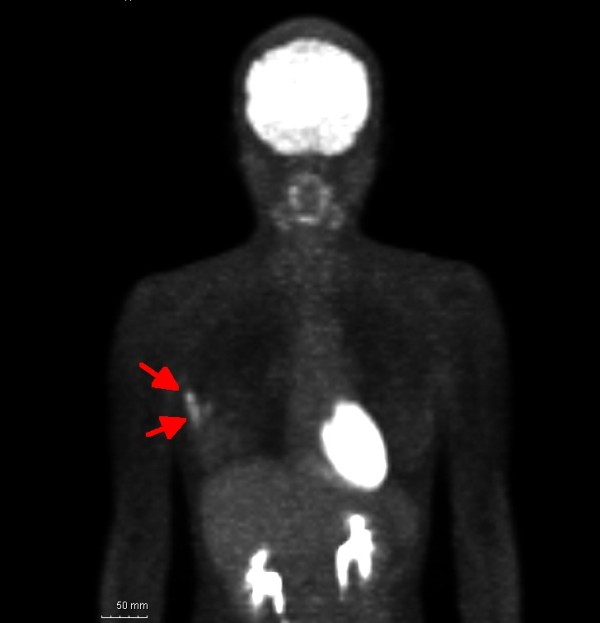
The arrow in this positron emission tomographic image indicates enhanced areas including the axillary mass and the lesion in the upper outer quadrant of the breast.

She was referred to the hematological department for evaluation of lymphadenopathy. PET was performed and not only the axillary region but also the outer upper quadrant of the breast were enhanced on the image (Figure 1). She was thus referred to our department for evaluation.

**Figure 2:**
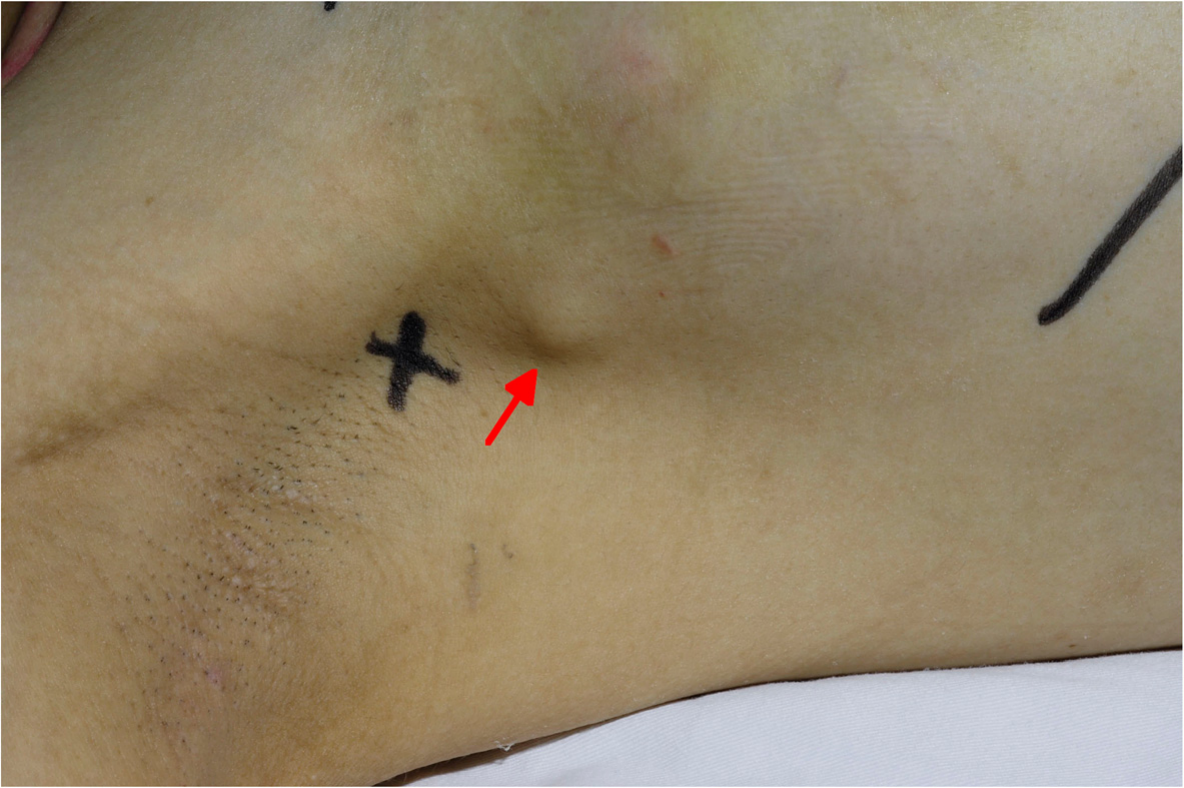
The arrow points to an axillary mass.

On physical examination, the patient had a 2-cm mass in her right axillary fossa (Figure 2). There were no overlying skin changes and no signs of an accessory nipple.

Her screening mammography showed no abnormal findings. However, spot mammography confirmed a round well-circumscribed high-density mass in the axillary area. Ultrasonography showed a 1.4-cm oval-shaped, well-circumscribed echoic mass corresponding to the axillary lesion on physical examination and mammography. There were no abnormal findings, however, in the vicinity of the tumor. No swollen lymph nodes were detected.

**Figure 3:**
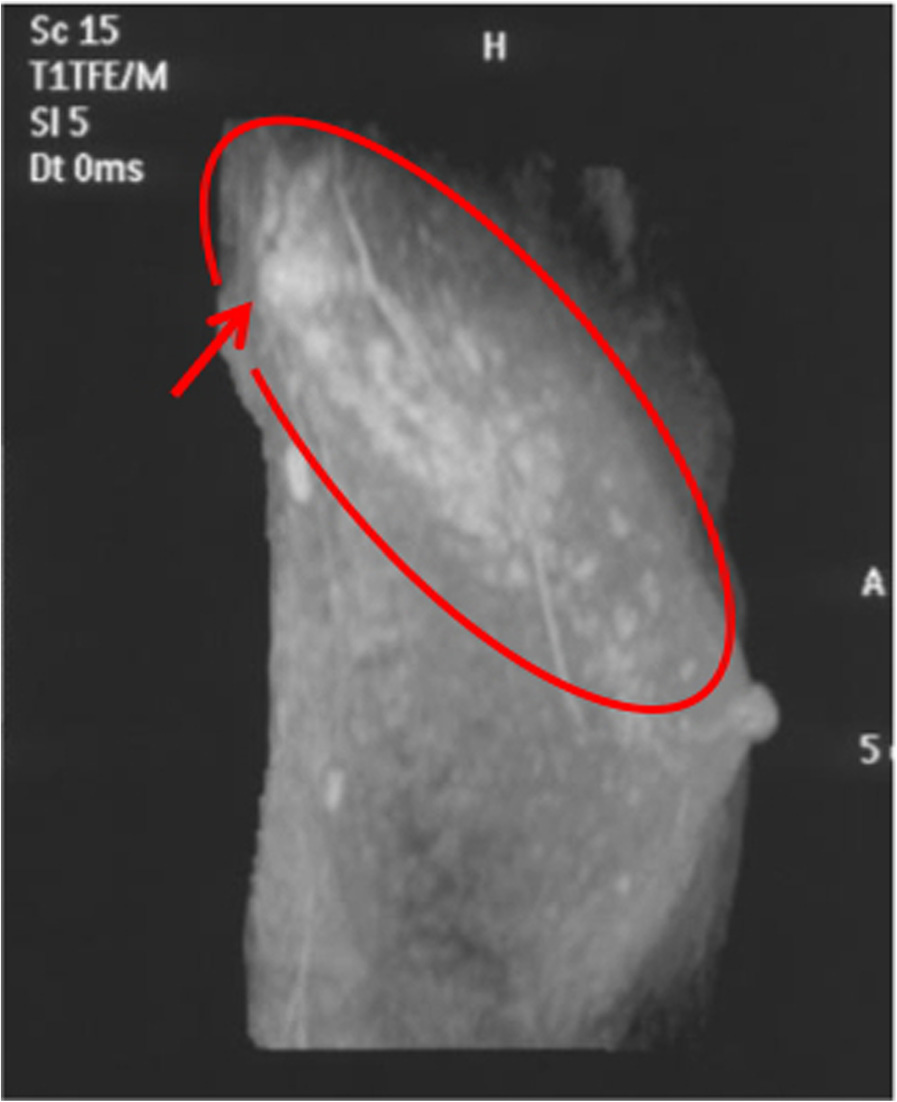
The circle in this breast magnetic resonance image indicates an enhanced area in the upper outer quadrant of the breast, and the arrow indicates the axillary tumor.

Enhanced MRI was performed and showed a segmentally enhanced area in the entire upper outer quadrant including the axillary mass (Figure 3). There were no abnormal findings in the contralateral side of the breast. After the enhanced MRI, we carried out a second-look ultrasound (US) examination. However we could not find any abnormal findings in the upper outer quadrant of the breast.

Core needle biopsy was preformed for the axillary mass and showed invasive ductal carcinoma. Subsequently, vacuum-assisted biopsy was performed for the upper outer quadrant mass, 2 cm away from the axillary mass towards the nipple without image guidance. Pathology revealed an intra-ductal carcinoma at this site.

According to these findings, we determined that the patient had breast cancer in the axillary tail of Spence with extended intra-ductal spread. We performed total mastectomy and sentinel node biopsy. The sentinel lymph nodes were negative.

**Figure 4:**
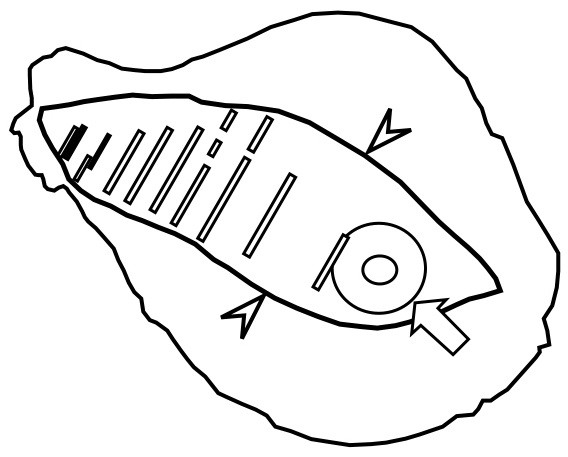
**Sketch of the surgical material.** The material was examined at intervals of 5 to 10 mm. The solid boxes indicate invasive lesions identified histologically; the blank boxes show intra-ductal lesions. The invasive lesions were continuous with the intra-ductal lesions. The white arrow indicates the alveolar and nipple, and the arrowheads show the removed skin.

Pathological examination of the surgical material revealed 1.9-cm invasive lesions accompanied by an 8.5-cm intra-ductal lesion. These lesions were continuous with each other (Figure 4). The characteristics of her invasive lesion were as follows: estrogen receptor (ER)-positive, progesterone receptor-positive, HER2-negative, 23% of Ki67, nuclear grade 3, and no sign of lymph-vascular invasion.

The patient received chemotherapy in the form of docetaxel and cyclophosphamide, followed by tamoxifen therapy. The patient is well 8 months post-operatively and has no signs of recurrence.

## Discussion

We report the case of an axillary mass, which was revealed to be breast cancer originating from the axillary tail of Spence. In cases of cancerous masses in the axillary area without any apparent primary breast tumors, there are a number of possible explanations. The clinical situation could be one of occult breast cancer, axillary tail breast cancer, ectopic breast cancer, or axillary metastasis from a non-breast organ.

Breast cancer in the axillary tail of Spence is extremely rare. Ampil *et al*. reported a frequency estimated at 0.1% [1]. Our particular case of this rare type of breast cancer has two unique characteristics. The first is that the tumor was located in the center of the axillary fossa. Many reported cases of this type of breast cancer describe the mass as located in the anterior fold of the axilla [2], or ‘encroaching partly on the mammary tail’ [3]. The axillary tail of Spence is of prime concern after prophylactic mastectomy and breast reconstruction as it represents remnant breast tissue left in place [2]. Our case demonstrates that complete removal of breast tissue in the axilla for prophylactic mastectomy is a difficult clinical problem.

The second characteristic of our case is that the newer imaging studies of enhanced MRI and PET study aided in preoperative diagnosis. Definitive diagnosis of breast cancer in the axillary tail requires microscopic examination, demonstrating that the breast cancer is continuous to the breast tissue in the upper outer quadrant. It is usually difficult to differentiate axillary tail breast cancer based on physical examination, mammography or ultrasonography.

Ectopic breast cancer is a rare diagnosis. Its frequency is estimated at 0.3% [4]. In a 7-week embryo, the mammary ridge, a band-line thickening of the epidermis, extends from the base of the forelimb to the hindlimb. Although the majority of the mammary line disappears shortly after its formation, a small portion in the thoracic region persists and penetrates the underlying mesenchyme, forming the mammary gland. Occasionally, fragments of the mammary line persist, forming polythelia [5]. The result is that accessory nipples can form along this mammary line. Accessory nipples occur most frequently in the axillary region, and more commonly overall in Asians as compared to Caucasians [6].

Ectopic breast tissue is divided into two categories, namely supernumerary breast and aberrant breast. Supernumerary breast has an organized duct system communicating with the overlying skin. Aberrant tissue is an island of breast tissue with no organized secretory system and has no relation to the overlying skin [6].

Histologic examination can aid in the diagnosis, identifying the presence of a histologic pattern of a primary breast carcinoma *in situ*, the presence of normal breast tissue in the vicinity of the tumor, and immunohistological characteristics such as ER and gross cystic disease fluid protein (GCDFP)-15. Management of primary ectopic breast carcinoma follows the normal breast cancer management guidelines [7].

Occult breast cancer with axillary lymph node metastasis is also rare, with frequencies reported from 0.12 to 0.67% [8]. Axillary lymph node metastasis can be the result of several primary tumors, including those from the breast, the gastrointestinal tract, the genitourinary tract, the skin, the thyroid, and the lung, as well as head and neck cancers [9]. It is therefore imperative that appropriate and sensitive imaging be carried out, including MRI and PET [10], with systemic, whole-body screening. Axillary metastases with an unknown primary source are known as occult breast cancers. It is recommended that occult breast cancer be treated in accordance with normal breast cancer management, with mastectomy being acceptable in most cases [8]. However, one third of cases of occult breast cancer show no lesions upon whole pathological breast examination [11].

In this case it was difficult to differentiate between primary breast cancer and metastatic lymph nodes with extranodal infiltration. However, we concluded that our patient had primary breast cancer rather than extranodal infiltration for the following reasons: there were no lymph node components around her invasive lesion, and no signs of extended lymph node metastasis were found in the axillary area.

MRI is highly sensitive for detecting breast cancer. MRI is useful for the screening for breast cancer in high-risk women compared to US and mammography [12,13]. Furthermore, MRI is cost-effective in screening BRCA1/2 mutation carriers [14]. MRI evaluation of the contralateral breast in women with recently diagnosed breast cancer is also useful [15]. Furthermore, MRI has become a useful modality for the management of occult breast cancer [16]. Breast MRI has reportedly identified primary breast lesions in more than two thirds of patients with occult breast cancer [17]. Our case has shown that MRI is also useful for diagnosing breast cancer in the axillary tail of Spence.

PET provided further opportunity to examine the right breast in our case. However, the utility of PET in the management of breast cancer patients has not been established. Incidental breast abnormalities found in PET examinations are rare, and the association of breast abnormalities with breast cancer has been reported as 37.5 to 56.7% [18,19]. High cost, as well as radiation exposure to the medical personnel and the patient, preclude the use of PET for breast cancer screening. Had this patient presented to our department initially she would not have received PET, but final management would have remained the same based on enhanced MRI findings.

## Conclusions

In conclusion, we have reported a case of breast cancer in the axillary tail of Spence. Preoperative use of breast MRI and PET aided in diagnosis of this rare case of breast cancer.

## Consent

Written informed consent was obtained from the patient for publication of this case report and any accompanying images. A copy of the written consent is available for review for the Editor-in-chief of this journal.

## Abbreviations

ER: Estrogen receptor; GCDFP: Gross cystic disease fluid protein; MRI: Magnetic resonance imaging; PET: Positron emission tomography; US: Ultrasound.

## Competing interests

The authors declare that they have no competing interests.

## Authors’ contributions

MO collected data and contributed to drafting the manuscript. KT drafted the manuscript. MI contributed to the histopathological analyses. TN, KN and ET helped to collect data. TO and YS contributed financial support. All authors read and approved the final manuscript.
